# Comparisons of front plate, percutaneous sacroiliac screws, and sacroiliac anterior papilionaceous plate in fixation of unstable pelvic fractures

**DOI:** 10.1097/MD.0000000000007775

**Published:** 2017-09-08

**Authors:** Ronghe Gu, Weiguo Huang, Lijing Yang, Huijiang Liu, Kegong Xie, Zonggui Huang

**Affiliations:** a Department of Orthopedics, The First People's Hospital of Nanning, Nanning; b Department of Orthopedics, Affiliated Hospital of Youjiang Medical University for Nationalities, Baise, Guangxi, China.

**Keywords:** percutaneous sacroiliac screw internal fixation, sacroiliac anterior papilionaceous plate, sacroiliac anterior plate fixation, unstable pelvic fracture

## Abstract

**Background:**

This observational study was aimed at comparing the clinical efficacy of sacroiliac anterior plate fixation (SAPF), sacroiliac anterior papilionaceous plate (SAPP), and percutaneous sacroiliac screw internal fixation (PSCIF) introduced for patients with unstable pelvic fracture.

**Methods:**

Seventy-eight patients with unstable pelvic fracture (Tile type B or C) were recruited. Twenty-six patients underwent SAPF, 26 underwent SAPP, and 26 underwent PSCIF. Matta scores were calculated to evaluate the reduction of pelvic fractures, and Majeed scores were applied for the assessment of functional recoveries after surgery. Other perioperative clinical indicators were also recorded, including operation time, bleeding status, length of incision, ambulation time, fracture healing time, and incision infection.

**Results:**

Total operation time of PSCIF was remarkably shorter than that of SAPF and SAPP (*P* < .05), and the bleeding volume of SAPF and SAPP group was almost 26∼29 times as high as that of PSCIF group (*P* < .05). Besides, SAPP resulted in significant blood loss compared with SAPF (*P* < .05), while SAPF resulted in significantly larger operative incision length than SAPP and PSCIF (*P* < .05). Moreover, patients’ stay time was prolonged in both SAPF and SAPP groups than in the PSCIF group (*P* < .05). Patients who received PSCIF exhibited significantly higher Matta and Majeed scores than those who received SAPF (all *P* < .05). Finally, SAPP was associated with fewer complications than SAPF, and complications were barely observed in the PSCIF group.

**Conclusion:**

PSCIF may be more appropriate for patients with unstable pelvic fractures in comparison with SAPP and SAPF. Besides, SAPP is likely to be more efficacious than SAPF especially for Tile C patients.

## Abstract 2

Supplemental Digital Content is available in the text

## 1 Introduction

Pelvic fracture is a serious trauma mainly caused by life-threatening external forces, such as motorbike accidents and falls from a great height.^[1]^ Unstable pelvic fracture, predominately caused by high-energy trauma, is characterized by the posterior pelvic fracture with partial or total displacement of lateral pelvis.^[2]^ It has been reported that the incidence of pelvic fracture is 2% to 8%,^[3]^ and the global mortality of pelvic fracture even stands as high as 33%.^[4,5]^ As the sacroiliac joint union acts as the main stable structure of posterior loop to bear the body weights, any damages to sacroiliac joint union could lead to the instability of pelvis ring,^[6]^ Although basic measures including pelvic harness, pelvic c-clamp, and external fixateur were taken in pelvic fracture cases,^[1]^ further treatments to reconstruct stable sacroiliac joint union should be done.

Common techniques that are applied to fix unstable pelvic fractures primarily include sacroiliac anterior plate fixation (SAPF), percutaneous sacroiliac screw internal fixation (PSCIF), and sacroiliac anterior papilionaceous plate (SAPP), etc. PSCIF and SAPF can be both useful treatment for unstable pelvic ring injuries^[7]^; however, PSCIF has been reported to outperform SAPF in treatment of unstable pelvic fractures with less invasive, less bleeding, less pain, and rapid recovery than SAPF.^[8]^ Besides, highly frequent complications manifest after SAPF, including the injury of lateral femoral cutaneous nerve and lumbosacral trunk, minor claudication, back pain, and sexual dysfunction.^[9,10]^ SAPF may lead to the deformity of posterior pelvic ring because it rarely takes the posterior pelvic ring into consideration. In contrast, PSCIF is the recommended approach for posterior lesion treatment.^[11]^ Screws used in PSCIF can be placed percutaneously once a satisfactory closed reduction is obtained,^[12]^ thus avoiding implant prominence, surface contamination, and skin damage that are usually observed in the application of sacral bars or plating.^[13]^ However, subjects undertaking PSCIF also have a high rate of screw malposition accompanied with nerve and vessel injuries. SAPP, on the contrary, emerges as a novel device to improve pelvic stability, and the technique might avert the impairment of the adjacent neurovascular structures.

To date, SAPF, PSCIF, and SAPP have not been simultaneously compared in managing unstable pelvic fractures. To identify the best surgery option for pelvic fracture, this study compared the three fixation devices based on operation time, perioperative bleeding status, length of incision, ambulation time, fracture healing time, incision infection status, Matta radiological scoring, Majeed functional grading scale, and incidence of complications.

## 2 Methods

### 2.1 Study design

This is an observational study. All patients with pelvic fractures referred to the First People's Hospital of Nanning, China, for surgical treatment from November 2010 and December 2015 were recruited. The study protocol was approved by the First People's Hospital of Nanning and the ethics committee of the First People's Hospital of Nanning in January 2016. All participants signed the informed consent. All patients were followed up after surgery for 1 year. Conventional radiographs were obtained and assessed by an experienced physician who did not participate in their surgeries.

### 2.2 Participants in observational analysis

One hundred patients were recruited initially, whereas 22 patients were excluded afterwards. Among the excluded, 16 did not meet the inclusion criteria and 6 declined to participate, leaving 78 patients as the study population. Among the 78 participants, 26 underwent SAPF, 26 underwent PSCIF, and 26 underwent SAPP.

### 2.3 Inclusion and exclusion criteria

The patients would be included if they were older than 18 years; had stable vital signs and stayed sober; obtained fine pulse of arteria dorsalis pedis; had no other severe medical diseases; were to receive treatment of surgery in less than 3 weeks; were without any surgical contradictions; and were with unstable pelvic ring.

Patients were excluded if their pelvic fractures occurred on tumorous bone; they had any surgical contraindications; they were complicated by extrapelvic bleeding; and they suffered from brain injury.

### 2.4 Preoperative procedures

The fracture type of every patient was classified using Tile classification, which divides pelvic ring fractures into A, B, and C types based on the degree of pelvic instability. A is stable, B is rotationally instable, and C is rotationally and vertically instable.^[14]^ All patients underwent computed tomography (CT) scan (3 mm in thickness) and diagnostic radiographing, and their fracture types were categorized. Also, a new CT scan (1 mm in thickness) and 3-D reconstruction were performed on patients at arrival to the hospital following the preoperative protocol. Postoperative anteroposterior (AP) and inlet-outlet views were also obtained. All the radiological assessments were done by a physician who did not take part in the treatment.

### 2.5 Surgery

Patients with unstable pelvic fracture were generally operated on the 5^th^ to 14^th^ day after trauma. A modified ilioinguinal approach was adopted for the treatment of the pelvic fracture. Reduction and fixation of sacroiliac joint injury were completed through lilac fossa, and nervus cutaneus femoris lateralis was protected carefully when they were exposed. Patients with general anesthesia were required to take the supine position. Supplement Figure 1, shows the conventional radiographs of SAPF, PSCIF, and SAPP.

#### 2.5.1 SAPF

The incision began from the anterior superior iliac spine and extended approximately 10∼15 cm along the crista iliaca into the backward muscular layer. The abdominal muscle was stripped from inside the white ilium, and the iliacus was bluntly dissected from under the periost. Iliacus and pelvic organs were retracted, and anterior sacroiliac ligaments were separated from the attachment sites of the ilium until the lateral border of sacroiliac joint and sacrum were exposed. Either reduction forceps or screws were inserted into the iliac nodes for the reduction of pelvic fractures. Then, the reconstruction plate or T-shape plate was placed over the fracture of sacroiliac joint and fixed with clamp screws.

#### 2.5.2 PSCIF

Subjects with minor displaced fractures all achieved closed reduction with the aid of C-shape arm. The needle point was determined in the third last point between the anterior and posterior superior iliac spines. An incision (length: 1∼2 cm) was created and continuously extended to iliac periosteum. A needle was inserted into S1 through iliac bone and sacroiliac joint in the direction that was parallel to the pelvic cross-section. If the fixation of fracture was unstable, a second iliosacral screw would be drilled into S2 about 1.5 cm below the fixation point.

#### 2.5.3 SAPP

SAPP was designed in the shape of hemi-butterfly with 6 tack holes featured by sliding compression. SAPPs were divided into 3 categories based on their lengths (large: 23 mm, medium: 20 mm, and small: 17 mm). All SAPPs share the same width of 10 mm. The plate (Watson Cor., Changzhou, China) was directly placed on the reduced sacroiliac joints. The inner part of the plate should be close to the sacral side of sacroiliac joint, and be placed right over the lateral part of the sacral wing. The pin hole in the front of the plate was managed 0.5 cm away from the margin of the sacrum wing. Two kirschner wires (diameter: 2.0 mm) were applied to temporarily fix the plate. Ultimately, 2 screws (Watson Cor.) were fixed on the sacrum, whereas 4 screws (Watson Cor.) were fixed on the ilia.

### 2.6 Postoperative treatment

Fractures were radiographed with X-ray to ensure that the reduction was successfully achieved. Antibiotics were given on the basis of the incision infection conditions. Routine thrombus prevention was performed. Patients without spinal fracture were encouraged to exercise in bed 24 hours after surgery. Patients underwent splenectomy or partial splenectomy based on the degree of splenic rupture. The chest injuries, including rib fracture, pneumothorax, hemothorax, were also handled. Patients with concurrent acetabular fracture were managed with open reduction internal fixation. Spinal pelvic fixation was performed for patients with sacrum fracture. Nerve root decompression was performed on the exposed S1 and S2 of lumbosacral nerve trunk. Transcatheter arterial embolization was performed to cope with presacral venous plexus rupture.

### 2.7 Follow-up and pelvic recovery assessment

Indicators including operation time, perioperative bleeding status, length of incision, ambulation time, fracture healing time, and incision infection status were recorded. Patients were followed up every 3 months within 1 year after operation, and twice a year in the next 1 year after surgery. Three physicians were designated to the follow-up of the patients to eliminate the subjective bias.

Matta scoring^[15]^ is based on postoperative radiograph, and the reduction of pelvic fractures was assessed as excellent if the separation was ≤4 mm; fine if the separation ranged from 4 to 10 mm; acceptable if the separation was between 11 and 20 mm; and poor if the separation was more than 20 mm.

Majeed scoring^[16]^ was utilized to assess the recovery of pelvic fractures. Majeed score system consists of endurance of pain (30 points), endurance of walking (20 points), endurance of sitting (10 points), endurance of sexual intercourse (4 points), and endurance of standing (36 points). Overall, the functional recovery would be evaluated as excellent if the overall score was ≥85 points; good if the score ranges from 70 to 85 points; acceptable if the score varies between 55 and 69 points; and poor if the score was less than 55 points.

### 2.8 Statistical analysis

Measurement data (x ± s) were analyzed using the *t* test or 1-way analysis of variance (ANOVA), if the assumption of normal distribution was satisfied. Otherwise, nonparametric test would be used. Counted data were assessed by the χ^2^ test. Age, sex ratio, and other potential confounders [e.g., body mass index (BMI), time from injury to operation, and so on] were adjusted. Sensitivity analyses have been conducted based on the above confounders to test the robustness of the findings. SPSS19.0 (IBM SPSS Statistics 19.0; IBM Co, Armonk, New York) was employed to carry out all statistical analyses and the difference was considered statistical significant when *P* < .05. PASS 11 software (NCSS Co, East Kaysville, Utah) performed power analysis and calculated sample sizes for this study.

## 3 Results

### 3.1 Preoperative baseline clinical characteristics of participants

Preoperative clinical characteristics of patients were recorded (Table 1). A total of 26 participants were included in each group and no significant difference were observed in terms of age (*P* *=* .834), gender (*P* *=* .874), BMI (*P* = .737), time from injury to the operation (*P* *=* .950), reasons of injuring (*P* *=* .849), and tile classification (*P* *=* .892).

**Table 1:**
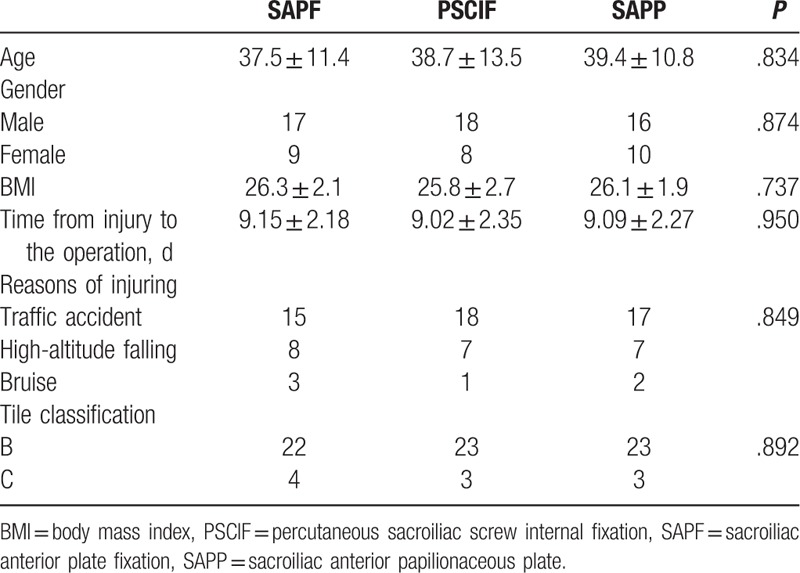
Preoperative clinical characteristics baseline of participants.

### 3.2 Perioperative clinical indicators

The average operation time of patients undergoing SAPF (118.5 ± 20.6 minutes) was significantly longer than those undergoing PSCIF (88.8 ± 14.0 minutes) and SAPP (106.6 ± 17.2 minutes), while PSCIF exhibited a significantly shorter operation time than SAPP (*P* < .05) (Table 2). The average bleeding volumes during surgery in SAPF (653.8 ± 144.5 mL) and SAPP groups (570.8 ± 127.5 mL) were both 25∼29 times as high as that of the PSCIF group (*P* < .05). Furthermore, SAPP significantly reduced the amount of blood loss compared with SAPF (*P* = .033). Stratified analysis by fracture severity indicated that pelvic fractures of type Tile B and C exhibited similar significant trends to the average with respect to operation time and blood loss (*P* < .05).

**Table 2:**
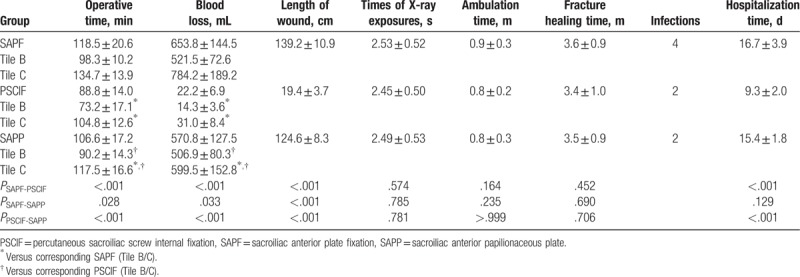
Comparisons of patients’ perioperative clinical indicators among SAPF, PSCIF, and SAPP groups.

In addition, the average operative incision lengths of SAPF (139.2 ± 10.9 cm) and SAPP (124.6 ± 8.3 cm) were significantly larger than that of PSCIF (19.4 ± 3.7 cm) (*P* < .05). Besides, the average incision length of SAPF was significantly larger than that of SAPP (*P* < .05). The stay-in-hospital time of patients in the SAPF and SAPP groups was significantly different from the PSCIF group (*P* < .05). However, the times of X-ray exposures, ambulation time, fracture healing time, and infections incidence were not different between the 3 groups.

### 3.3 Matta score

In terms of pelvic function recovery, there appeared to be no significant difference in the score of patients between the SAPF (excellent: 12; good: 5; acceptable: 4; poor: 5) and SAPP groups (excellent: 15; good: 8; acceptable: 3; poor: 0) (Table 3). Apart from that, there was no significant difference in the score of patients between the PSCIF (excellent: 16; good: 9; acceptable: 1; poor: 0) and SAPP groups (excellent: 15; good: 8; acceptable: 3; poor: 0) (*P* > .05). By contrast, patients with PSCIF exhibited evidently better favorable recovery than SAPF (*P* = .037).

**Table 3:**
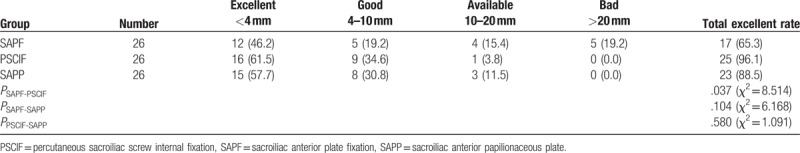
Comparison of postoperative Matta scores (n, %) among SAPF, PSCIF, and SAPP groups.

### 3.4 Majeed score

It is summarized in Table 4 that PSCIF (excellent: 14; good: 10; acceptable: 2; poor: 0) exhibited more favorable prognosis than SAPF (excellent: 6; good: 11; acceptable: 8; poor: 1) (*P* = .049), but the prognosis difference between the SAPF (excellent: 6; good: 11; acceptable: 8; poor: 1) and SAPP group (excellent: 13; good: 9; acceptable: 4; poor: 0) as well as that between the PSCIF and SAPP group were insignificant (*P* > .05).

**Table 4:**
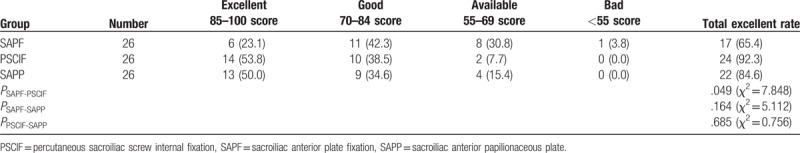
Comparison of postoperative Majeed scores (n, %) among SAPF, PSCIF, and SAPP groups.

### 3.5 Postoperative complications

The incidence of during-operation and follow-up complications between SAPP and SAPF was not significant (all *P* > .05) (Table 5). Of note, none of the complications were observed in patients undergoing PSCIF during or after the surgery. Compared with SAPP or SAPF group, PSCIF group showed no significant difference.

**Table 5:**
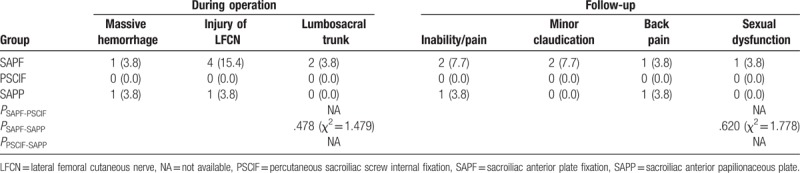
Comparisons of postoperative complications (n, %) among SAPF, PSCIF, and SAPP groups.

## 4 Discussion

As a main portion of the pelvis stable structure, sacroiliac complex is an indispensable structure of the human bone, as it accommodates the intrapelvic organs and props up both trunks and spine. Fractures of sacroiliac joints usually occur because of external violence. As pelvic fracture reduces one's mobility, rebuilding the structural stability of sacroiliac joint complex has been considered as an effective strategy. Both conservative exterior fixation and surgical intervention have been reported to effectively manage pelvic fracture.^[17–20]^ Nevertheless, conservative exterior fixation such as pelvic band, pelvic splint, and plaster external fixation usually takes longer time to recover from the fracture and may result in unexpectedly high mortality rate.^[15,21–23]^ As a result, surgical treatments that facilitate the recovery process and reduce the incidence of complications have been widely advocated, including sacrum plate fixation, SAPF, PSCIF, SAPP, and fixation of the spine pelvis nail rod system.^[7,23]^ Appropriate surgical methods are chosen to best fit in the fixation devices considering the fracture type and severity.^[24]^ Thus, this study was carried out to compare the tolerance and efficacy of SAPF, PSCIF, and SAPP in managing Tile B/C unstable pelvic fractures.

In accordance with our investigation, SAPF appeared to be the most time-consuming approach for managing patients with unstable pelvic fractures in comparison with PSCIF and SAPP. Moreover, SAPF exhibited the highest level of blood loss along with the largest wound length. Therefore, when the T-shape board is implanted into the subjects undergoing SAPF, the complex anatomical structures should be prudently examined, as iatrogenic injury may occur, and damages on the 4th and 5th lumbar nerve are usually observed clinically when nerves (length: >1–1.5 cm) from the sacroiliac joint were stripped.^[25]^ Although SAPP also requires the implantation of a steel plate, its simplified operation process usually takes less time in placing the steel plate and thus blood loss can be reduced. PSCIF appears to have the least harm, as only a small incision in the rear is required in the operation. However, there is no significant difference in ambulation time or fracture healing time among the above 3 approaches, suggesting that they might have similar efficacy in managing pelvic fracture, similar with the discovery of Morris et al.^[26]^

The superiority of PSCIF over SAPF with respect to Matta and Majeed rating may be explained by the fact that PSCIF mainly relies on the sacroiliac screw that is firmly fixed through sacroiliac joint in S1.^[27]^ The central location of PSCIF is usually near the fracture or dislocation area, contributing to more effective biomechanics and larger steel plate intensity than SAPF. However, Yu et al^[28]^ found no significant difference in the Majeed score between SAPF and PSCIF treatments (χ^2^ = 1.004, *P* > .05). This inconsistency may result from differences in the study cohorts and the operation conditions. Moreover, SAPP appears not to be significantly superior over SAPF in terms of Matta/Majeed ratings of patients, but SAPP could adapt to the irregular shape of sacrum and ilium than SAPF. As suggested by the observed during-surgery and after-surgery complications, SAPP was associated with fewer complications than SAPF. This is probably because that SAPP is able to fix sacroiliac joints on an entire plane with more biomechanical stability. Nonetheless, rarely any complications were observed in patients treated with PSCIF, which may be attributed to the smaller incisions PSCIF needed, resulting in the reduced risk of damage on certain crucial curves.^[29–31]^

However, there are still some limitations in our study. Only 78 patients were investigated and the sample size is comparatively small. Besides, age is an important factor that influences the treatment and prognosis of pelvic fractures, suggesting us that relevant researches are needed to investigate the correlation between the age and treatments. Besides, until now, pelvic fracture management is still challenging, as most management approaches are invasive. Hence, noninvasive treatment strategies are always encouraged.

Generally, SAPF, PSCIF, and SAPP are valid remedy options for managing unstable pelvic ring injuries. Our study suggested that SAPP might be more efficacious than SAPF for Tile C patients given that SAPP can simplify the operation process, shorten the time to place steel plate, and facilitate the rotating shift. Furthermore, PSCIF was a moderate percutaneous fixation with a minimum wound and it was reported to be efficacious in treating sacrum fractures and sacroiliac arthrosis split.

## Supplementary Material

Supplemental Digital Content

## References

[1] Scheyerer MJ Zimmermann SM Osterhoff G Anterior subcutaneous internal fixation for treatment of unstable pelvic fractures. BMC Res Notes 2014;7:133. 24606833 10.1186/1756-0500-7-133 PMC3975274

[2] Zhao JX Zhao Z Zhang LC A computer aided measurement method for unstable pelvic fractures based on standardized radiographs. BMC Med Imaging 2015;15:41. 26423682 10.1186/s12880-015-0084-x PMC4588254

[3] Kanezaki S Miyazaki M Notani N Clinical presentation of geriatric polytrauma patients with severe pelvic fractures: comparison with younger adult patients. Eur J Orthop Surg Traumatol 2016;26:885–90. 27448282 10.1007/s00590-016-1822-7

[4] Smith W Williams A Agudelo J Early predictors of mortality in hemodynamically unstable pelvis fractures. J Orthop Trauma 2007;21:31–7. 17211266 10.1097/BOT.0b013e31802ea951

[5] Giannoudis PV Pape HC Damage control orthopaedics in unstable pelvic ring injuries. Injury 2004;35:671–7. 15203307 10.1016/j.injury.2004.03.003

[6] Giannoudis PV Fachgebiete HMA Practical Procedures in Elective Orthopaedic Surgery. London: Springer; 2012.

[7] Choy WS Kim KJ Lee SK Anterior pelvic plating and sacroiliac joint fixation in unstable pelvic ring injuries. Yonsei Med J 2012;53:422–6. 22318833 10.3349/ymj.2012.53.2.422 PMC3282962

[8] Li CL Clinical comparative analysis on unstable pelvic fractures in the treatment with percutaneous sacroiliac screws and sacroiliac joint anterior plate fixation. Eur Rev Med Pharmacol Sci 2014;18:2704–8. 25317806

[9] Mason WT Khan SN James CL Complications of temporary and definitive external fixation of pelvic ring injuries. Injury 2005;36:599–604. 15826617 10.1016/j.injury.2004.11.016

[10] Zhou KH Luo CF Chen N Minimally invasive surgery under fluoro-navigation for anterior pelvic ring fractures. Indian J Orthop 2016;50:250–5. 27293284 10.4103/0019-5413.181791 PMC4885292

[11] Langford JR Burgess AR Liporace FA Pelvic fractures: part 2. Contemporary indications and techniques for definitive surgical management. J Am Acad Orthop Surg 2013;21:458–68. 23908252 10.5435/JAAOS-21-08-458

[12] Keating JF Werier J Blachut P Early fixation of the vertically unstable pelvis: the role of iliosacral screw fixation of the posterior lesion. J Orthop Trauma 1999;13:107–13. 10052785 10.1097/00005131-199902000-00007

[13] Miller AN Routt ML Jr Variations in sacral morphology and implications for iliosacral screw fixation. J Am Acad Orthop Surg 2012;20:8–16. 22207514 10.5435/JAAOS-20-01-008

[14] Tile M Acute pelvic fractures: I. Causation and classification. J Am Acad Orthop Surg 1996;4:143–51. 10795049 10.5435/00124635-199605000-00004

[15] Matta JM Tornetta P 3rd Internal fixation of unstable pelvic ring injuries. Clin Orthop Relat Res 1996 129–40. 10.1097/00003086-199608000-00016 8769444

[16] Majeed SA Grading the outcome of pelvic fractures. J Bone Joint Surg Br 1989;71:304–6. 2925751 10.1302/0301-620X.71B2.2925751

[17] Rommens PM Hessmann MH Staged reconstruction of pelvic ring disruption: differences in morbidity, mortality, radiologic results, and functional outcomes between B1, B2/B3, and C-type lesions. J Orthop Trauma 2002;16:92–8. 11818803 10.1097/00005131-200202000-00004

[18] Suzuki T Shindo M Soma K Long-term functional outcome after unstable pelvic ring fracture. J Trauma 2007;63:884–8. 18090021 10.1097/01.ta.0000235888.90489.fc

[19] Nepola JV Trenhaile SW Miranda MA Vertical shear injuries: is there a relationship between residual displacement and functional outcome? J Trauma 1999;46:1024–9. discussion 1029–1030. 10372618 10.1097/00005373-199906000-00007

[20] Smith W Shurnas P Morgan S Clinical outcomes of unstable pelvic fractures in skeletally immature patients. J Bone Joint Surg Am 2005;87:2423–31. 16264117 10.2106/JBJS.C.01244v

[21] Stocks GW Gabel GT Noble PC Anterior and posterior internal fixation of vertical shear fractures of the pelvis. J Orthop Res 1991;9:237–45. 1992074 10.1002/jor.1100090212

[22] Chen PH Hsu WH Li YY Outcome analysis of unstable posterior ring injury of the pelvis: comparison between percutaneous iliosacral screw fixation and conservative treatment. Biomed J 2013;36:289–94. 24385071 10.4103/2319-4170.112757

[23] Henderson RC The long-term results of nonoperatively treated major pelvic disruptions. J Orthop Trauma 1989;3:41–7. 2523480 10.1097/00005131-198903010-00008

[24] Heydemann J Hartline B Gibson ME Do transsacral-transiliac screws across uninjured sacroiliac joints affect pain and functional outcomes in trauma patients? Clin Orthop Relat Res 2016;474:1417–21. 26472585 10.1007/s11999-015-4596-z PMC4868165

[25] Leighton RK Waddell JP Techniques for reduction and posterior fixation through the anterior approach. Clin Orthop Relat Res 1996 115–20. 10.1097/00003086-199608000-00014 8769442

[26] Morris SA Loveridge J Smart DK Is fixation failure after plate fixation of the symphysis pubis clinically important? Clini Orthop Relat Res 2012;470:2154–60. 10.1007/s11999-012-2427-z PMC3392398 22707071

[27] Routt ML Jr Simonian PT Mills WJ Iliosacral screw fixation: early complications of the percutaneous technique. J Orthop Trauma 1997;11:584–9. 9415865 10.1097/00005131-199711000-00007

[28] Yu C Comparison on Efficacy of Fixation of Unstable Pelvic Fracture Between Front Plate and Sacroiliac Screws. Dalian, China: Dalian Medical University; 2014.

[29] Riehl J Widmaier J A simulator model for sacroiliac screw placement. J Surg Educ 2012;69:282–5. 22483125 10.1016/j.jsurg.2011.10.012

[30] Giannoudis PV Chalidis BE Roberts CS Internal fixation of traumatic diastasis of pubic symphysis: is plate removal essential? Arch Orthop Trauma Surg 2008;128:325–31. 17713770 10.1007/s00402-007-0429-1

[31] Lange RH Hansen ST Jr Pelvic ring disruptions with symphysis pubis diastasis. Indications, technique, and limitations of anterior internal fixation. Clin Orthop Relat Res 1985 130–7. 4064397

