# Supplementary material for: Comparisons of front plate, percutaneous sacroiliac screws, and sacroiliac anterior papilionaceous plate in fixation of unstable pelvic fractures
Source: Medicine (Baltimore). 2017 Sep 8;96(36):e7775. doi: 10.1097/MD.0000000000007775 (PMC6392970; doi:10.1097/MD.0000000000007775)

**SUPPLEMENT FIGURE LEGENDS**

**Supplement Figure 1. Pre- and postoperative X-ray examinations of pelvic fracture patients with different treatment.** SAPF: sacroiliac anterior plate fixation, SAPP: sacroiliac anterior papilionaceous plate, PSCIF: percutaneous sacroiliac screw internal fixation.


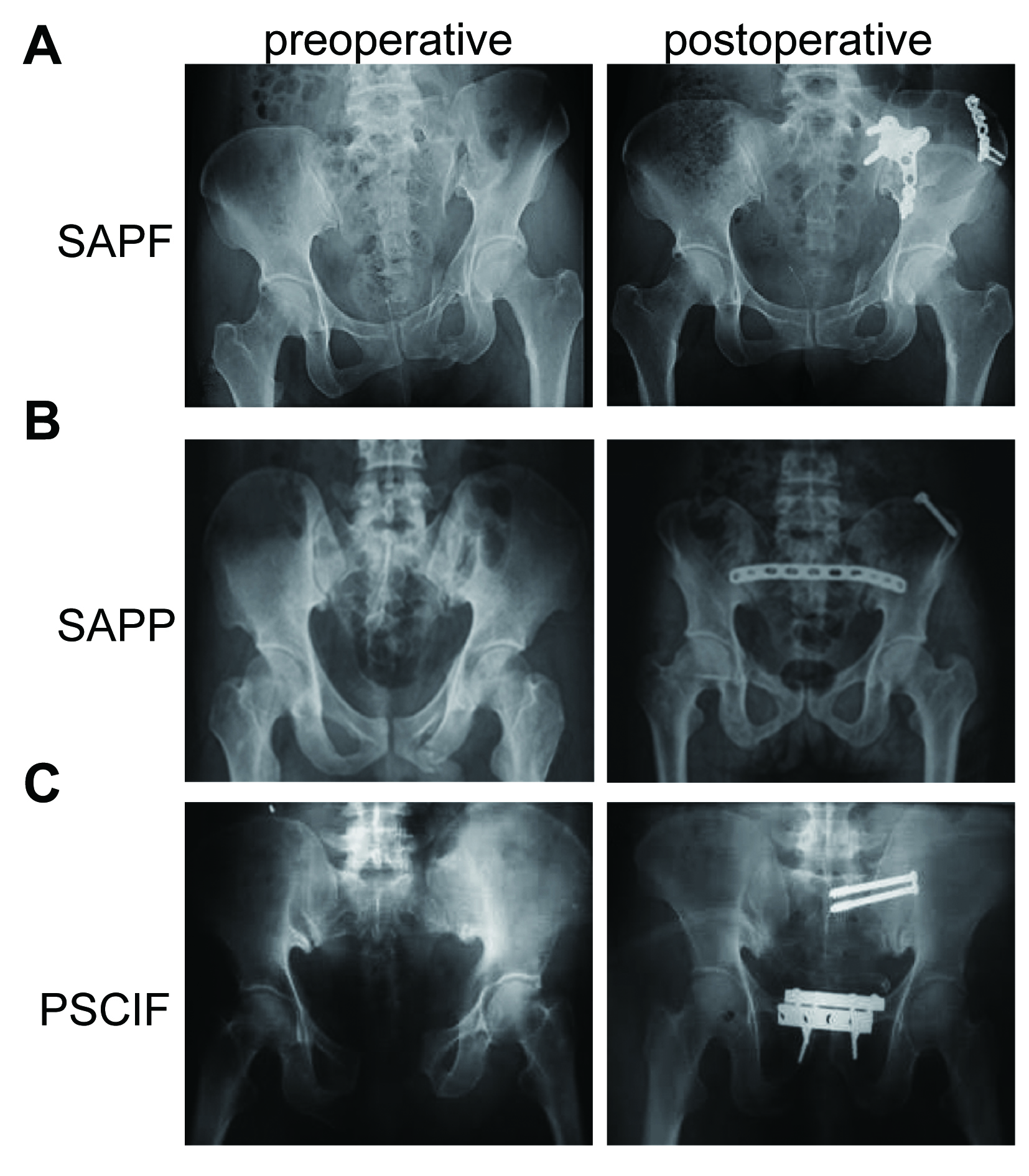

Supplement: Supplemental Digital Content [file medi-96-e7775-s001.doc]
